# Use of a Blockade-of-Binding ELISA and Microneutralization Assay to Evaluate Zika Virus Serostatus in Dengue-Endemic Areas

**DOI:** 10.4269/ajtmh.19-0270

**Published:** 2019-08-06

**Authors:** Eduardo J. M. Nascimento, Matthew I. Bonaparte, Ping Luo, Timothy S. Vincent, Branda Hu, James K. George, Germán Áñez, Fernando Noriega, Lingyi Zheng, James W. Huleatt

**Affiliations:** 1 Global Clinical Immunology, Sanofi Pasteur, Swiftwater, Pennsylvania;; 2 Global Clinical Sciences, Sanofi Pasteur, Swiftwater, Pennsylvania

## Abstract

Zika virus (ZIKV) serological diagnostics are compromised in areas where dengue viruses (DENV) co-circulate because of their high levels of protein sequence homology. Here, we describe the characterization of a Zika blockade-of-binding ELISA (Zika BOB) and a Zika microneutralization assay (Zika MN) for the detection of ZIKV nonstructural protein 1 (NS1)–specific antibodies and ZIKV neutralizing antibodies, respectively. Zika BOB and Zika MN cutoffs were established as 10 and 100 endpoint titers, respectively, using samples collected pre- and post-virologically confirmed ZIKV infection from subjects living in DENV-endemic areas. Specificity of the assays was equally high, whereas sensitivity of Zika BOB was lower than that of Zika MN, especially in samples collected > 6 months post-infection. Immunosurveillance analysis, using combined results from both Zika BOB and Zika MN, carried out also in DENV-endemic regions in Colombia, Honduras, Mexico, and Puerto Rico before (2013–2014) and after (2017–2018) ZIKV introduction in the Americas suggests unapparent ZIKV seroprevalence rates ranged from 25% to 80% over the specified period of time in the regions investigated.

## INTRODUCTION

Whereas Zika virus (ZIKV) was initially detected in 2013 in the Americas,^1^ its first apparent outbreak with widespread clinical manifestation was detected in Brazil in early 2015. Since then, ZIKV rapidly disseminated with high attack rates^2^ throughout South and Central America and the Caribbean, especially in areas where the seroprevalence of dengue viruses (DENV) is high.^3–6^ Those outbreaks were linked with neurological disorders in adults^7,8^ and devastating neurological consequences in the children of mothers infected during pregnancy.^9^ Both DENV and ZIKV are members of the genus *Flavivirus*, which are transmitted by the same mosquito vector (predominantly *Aedes aegypti*) and share a high degree of protein identity.^10^ The factors contributing to the severity of clinical outcomes following ZIKV infection are not fully understood, although a potential role of DENV (and other arboviruses) co-transmission has been proposed.^11^ Several reports have suggested that antibodies directed to the DENV envelope protein (E) may enhance the infection of phagocytes by ZIKV in vitro,^12,13^ although such an association has not been confirmed either clinically or epidemiologically.^14,15^ In addition, the high homology in the primary amino acid sequence between DENV and ZIKV poses a significant challenge in defining virus serostatus in areas of endemic transmission where these viruses co-circulate,^13,16,17^ as serum antibodies from DENV-immune subjects cross-react with ZIKV antigens and vice versa.^18–22^ Neutralizing antibody titers assessed by plaque-reduction neutralization test (PRNT) have successfully been used to differentiate ZIKV from DENV infections, especially at the late convalescent stage of infection, despite a persistent degree of cross-reactivity after recent exposure to both viruses.^23^ In addition, low-throughput, longer turnaround time, and the need for experienced and highly trained personnel make PRNT a challenge for high-demand clinical testing.^24^ On the other hand, most of the in-house and commercially available immunoassays are based on detection of antibodies to the two major targets of immune responses following ZIKV infection, the E and non-structural 1 (NS1) proteins, and have limited specificity,^13,18,21,22^ with the exception of a ZIKV NS1 IgG3 ELISA^2^ and Zika NS1 blockade-of-binding (BOB) ELISA.^19,25^ The BOB ELISA is a competitive ligand-binding assay and relies on the ability of serum antibodies to block the binding of a highly specific monoclonal antibody (mAb) to an antigen adsorbed on a microtiter ELISA plate.^26^ This approach has been used with high specificity to detect antibodies against many viruses, including Crimean–Congo hemorrhagic fever virus,^27^ foot-and-mouth disease virus,^28^ bluetongue virus,^29^ and West Nile virus (WNV).^30^

We carried out a proof-of-concept study to determine Zika serostatus using a Zika NS1 BOB ELISA (Zika BOB) and a high-throughput colorimetric Zika microneutralization assay (Zika MN) in samples collected in DENV-endemic areas affected by ZIKV outbreaks in 2016. In addition, the combination of results of both methods was used to estimate unapparent ZIKV exposure (recent and remote) in Colombia, Honduras, Mexico, and Puerto Rico before (2013–2014) and after (2017–2018) the introduction of ZIKV to the Americas.

## MATERIALS AND METHODS

### Ethics statement.

The trial protocols were approved by all relevant ethics review boards, and parents or guardians provided written informed consent and older children provided written informed assent before participation in the study, in accordance with local regulations. All data were anonymized such that no patient identifiers were present in the data files received for analysis.

### Recombinant proteins.

Recombinant NS1 proteins from the following flaviviruses were obtained commercially (Native Antigen Company, Oxfordshire, United Kingdom): Zika virus (ZIKV, strain Suriname, which circulated in the Americas in 2015); DENV serotypes 1 (strain Nauru/Western Pacific/1974), 2 (strain Thailand/16681/84), 3 (strain Sri Lanka D3/H/IMTSSASRI/2000/1266), and 4 (strain Sri Lanka D3/H/IMTSSA-SRI/2000/1266); yellow fever virus (YFV; strain 17D); Japanese encephalitis virus (JEV; strain SA-14 ); tick-borne encephalitis virus (TBEV; strain Neudoerfl); West Nile virus (WNV; strain NY99); and Usutu virus (USUV; strain Vienna 2001).

### Unrelated proteins.

Non-recombinant *Bordetella pertussis* toxin (Marcy L’Etoile, France) and *Clostridium difficile* toxin B (Swiftwater) were manufactured by Sanofi Pasteur and were used as unrelated antigens in specificity (competition) experiments.

### Zika NS1 BOB ELISA procedure.

**Figure 1.**
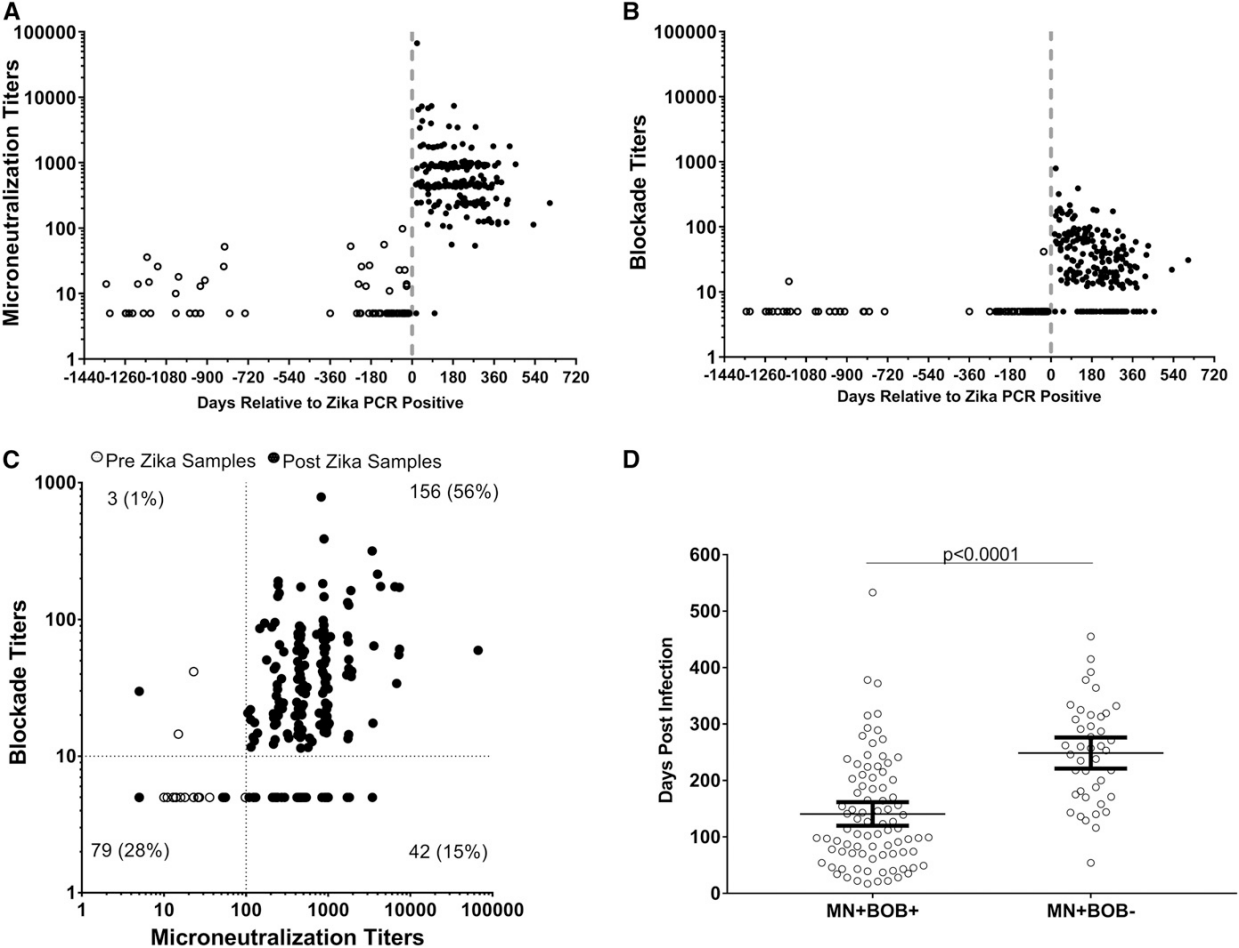
Kinetics of microneutralization and Zika blockade-of-binding (BOB) ELISA activity in Zika virus (ZIKV) (VCZ) and dengue virus (VCD) virologically confirmed samples (**A**) Microneutralization (MN) titers and (**B**) Zika nonstructural protein 1 BOB ELISA titers in longitudinal VCZ samples from before and after ZIKV infection. For plots (**A**) and (**B**), time 0 represents the index sample obtained when virus infection was initially detected by ZIKV RT-PCR. Open circle represents samples collected before ZIKV infection. Black circle represents samples collected post-ZIKV infection. A value of 5 (1/2 lower limit of quantification, LLOQ) was assigned to samples below the LLOQ of 10. (**C**) Two-dimensional dot plot comparing Zika MN and Zika BOB titers on pre- and post-Zika samples. Percentage of the total number of samples (pre- and post-Zika) evaluated is shown in each quadrant. (**D**) Comparison of collection time relative to the identification of ZIKV infection on Zika MN–positive samples. Collection time in days were compared in MN-positive and BOB-positive (MN+ BOB+) (*n* = 156) samples and MN-positive and BOB-negative (MN+ BOB−) (*n* = 42) samples. Time post-infection is shown for each individual sample and line, and error bars represent average and 95% CIs. The average days post-infection was compared between the groups using unpaired Student’s *t*-test.

Zika NS1 BOB ELISA measures the levels of serum antibodies that block the binding of a highly specific mAb to Zika NS1 as described as follows: Thermo Immulon 2HB (Thermo Scientific, Waltham, MA) 96-well flat-bottom microtiter plates were coated with ZIKV NS1 in carbonate/bicarbonate buffer (pH 9.6 ± 0.1) overnight at 4°C. The plates were washed with 0.01 M phosphate-buffered saline (PBS) with 0.05% Tween 20 (PBS-T; Hyclone Laboratories, Logan, UT) and blocked with PBS-T supplemented with 1% (v/v) goat normal serum (1% GNS; Gibco Laboratories, Gaithersburg, MD) for 45 ± 5 minutes at 21°C. The plates were washed with PBS-T, then 2-fold serially diluted human samples and internal quality controls (IQC; human samples obtained commercially from ZIKV-exposed individuals in Colombia [ABO Pharmaceuticals, San Diego, CA]) in 1% GNS were supplemented with a pool of DENV NS1 from all four DENV serotypes at 0.5 μg/mL, and incubated for 60 ± 5 minutes at 21°C to reduce cross-reactivity by DENV-specific antibodies to ZIKV NS1–coated plates. A solution containing ZIKV NS1–specific mouse mAb, clone 1F11.B7.A2.F9 (Native Antigen Company; see Supplemental Table 1 and Figure 1A for binding specificity analysis), at 0.5 μg/mL prepared in 1% GNS was immediately pipetted on top of the human samples, mixed, and incubated for 10 ± 5 minutes at 21°C. The plates were washed with PBS-T and incubated for 60 ± 5 minutes at 21°C with peroxidase-conjugated F(ab′)_2_ goat anti-mouse IgG Fcɣ fragment (Jackson ImmunoResearch Laboratories, West Grove, PA) prepared in 1% GNS. The plates were washed with PBS-T and developed with SureBlue Reserve tetramethylbenzidine (TMB) microwell peroxidase substrate (SeraCare, Milford, MA) for 30 ± 2 minutes at 21°C. The reaction was stopped with 1N hydrochloric acid (Fisher Scientific, Fair Lawn, NJ) and the plates were read in a SpectraMax 384 (Molecular Devices, Sunnyvale, CA) microplate reader at 450 nm (650 nm as the reference wavelength) using SoftMax Pro software version 6.5.1 (Molecular Devices). For best assay precision, blockade titers are calculated by plotting and performing linear regression fit of the optical density of two dilution points (immediately below and above the signal cutoff) with SoftMax Pro software and reported as continuous dilution of the sample that inhibits 50% of the binding of the mAb, as shown in Supplemental Figure 2. The assay acceptance criteria include three IQCs as well as the conjugate blank, mAb signal, and percentage of coefficient of variance (%CV) in each plate for data validity (Supplemental Table 2).

### Zika virus MN procedure.

Zika virus MN measures ZIKV neutralizing antibody titers using a colorimetric readout as follows: in 96-well tissue culture microplates (Corning Life Sciences, Corning, NY), sera to be tested were diluted 1:5 with minimum essential medium (Gibco Laboratories), supplemented with 5% fetal bovine serum (Hyclone Laboratories), 10 mM HEPES, 20 mM L-glutamine, 100 units/mL of penicillin, 100 μg/mL of streptomycin, and 0.25 μg/mL of amphotericin B (HEPES, glutamine, and antibiotics from Gibco Laboratories), and 2-fold serial dilutions were performed using 50 μL aliquots across the rows of the plate. Six hundred plaque-forming units (PFU) per well of ZIKV strain PRVABC59 (VR-1843, American Type Culture Collection [ATCC], Rockville, MD) challenge dose (in 50 μL) was mixed with the serially diluted sera and allowed to neutralize virus infectivity for 1 hour at 37°C. A separate virus titration plate was prepared to determine the 50% tissue culture infective dose (TCID_50_). For this, eight replicates of 3-fold serial dilutions of the working concentration of virus (600 PFU in 50 μL) were performed across the rows of a 96-well tissue culture plate and the plate was incubated at 37°C for 1 hour. Following neutralization, a 100-μL aliquot containing 80,000 cells of a freshly prepared suspension of Vero cells (CCL-81, ATCC) was added to each well of the plates. The plates were incubated for 4 days at 37°C with 5% CO_2_ and humidity, then washed and fixed with 80% acetone (Thermo Fisher Scientific), followed by blocking with 5% nonfat milk (Carnation Company, Los Angeles, CA) in PBS-T, and incubated with an anti-pan *Flavivirus* mAb, HB112-4G2 (Biotem Inc., Apprieu, France). The plates were washed with PBS-T and incubated with horseradish-conjugated goat anti-mouse IgG (Jackson ImmunoResearch Laboratories) for 1 hour. The plates were washed with PBS-T and developed with the TMB microwell peroxidase substrate system (SeraCare) for 45 minutes at 20°C. The reaction was stopped with 2N sulfuric acid (Macron Chemicals, Center Valley, PA) and the plates were read in a SpectraMax 384 (Molecular Devices) microplate reader at 450 nm (650 nm as the reference wavelength) using SoftMax Pro software version 6.5.1 (Molecular Devices). The serum titers were determined by calculating the sample dilution that neutralized 50% of the maximum signal using SoftMax Pro software. The assay acceptance criteria include three IQCs and the TCID_50_ in each assay run for data validity (Supplemental Table 2).

### Dengue NS1 IgG ELISA procedure.

Dengue NS1 IgG ELISA is an immunoassay to quantify IgG to DENV NS1 and is described elsewhere.^20^ Briefly, 96-well flat-bottom microtiter plates were coated with pooled DENV NS1 from all four DENV serotypes (at 1:1:1:1 ratio) in carbonate/bicarbonate buffer (pH 9.6) and left overnight at 4°C. The plates were washed with PBS-T and then blocked with PBS-T supplemented with 1% GNS for 45 minutes at 21°C. After washing, 2-fold serially diluted human samples and IQCs (described earlier) in 1% GNS were added and incubated for 60 minutes at 37°C. The plates were washed with PBS-T and incubated for 60 minutes at 37°C with an anti-human IgG detection antibody, developed with a TMB substrate, and read as described earlier.

### Zika immunoassay characterization parameters.

Assay performance evaluation of both Zika NS1 BOB ELISA and ZIKV MN was carried out based on the International Conference for Harmonization Harmonized Tripartite Guideline^31^ and is shown in the Supplemental Material.

### Virologically confirmed Zika (VCZ) and dengue (VCD) samples.

Longitudinal ZIKV antibody-positive human serum samples were obtained from febrile (≥ 38°C for at least 2 days) subjects who had a VCZ by real-time reverse transcriptase–polymerase chain reaction (qRT-PCR; ARUP Laboratories, Salt Lake City, UT). Samples available before and after the qRT-PCR diagnosis were used for evaluation of specificity and sensitivity, respectively, of Zika BOB and Zika MN. Subjects had participated in the CYD15 Phase III efficacy clinical trial of the Sanofi Pasteur CYD-TDV vaccine (ClinicalTrials.gov Identifier: NCT01374516) in Colombia, Honduras, Mexico, and Puerto Rico. The RT-qPCR, considered the gold standard for ZIKV diagnosis, is run on the QuantStudio 12K Flex instrument (Life Technologies, Carlsbad, CA). The assay uses the 2× custom multiplex 1-step RT-qPCR master mix from Quanta Biosciences and primers and probes supplied by ELITech. Zika is detected in the fluorescein amidite channel using primers and probes that are specific to the non-structural protein 3 (NS3)–encoding gene and generate an amplicon of 114 base pairs. A noncompetitive RNA internal control (ms2 phage) is added to the lysis buffer and monitored for RNA extraction and qRT-PCR inhibition.

To determine false positivity rates of Zika BOB, samples were also obtained from VCD infections from countries cited previously from the CYD15 study in addition to the Philippines (CYD14 phase III clinical trial; ClinicalTrials.gov Identifier: NCT01373281). Day “0” represents the day on which the viral infection was confirmed based on the qRT-PCR methods described previously.^32^

For all samples analyzed, time of infection was determined relative to the date of obtaining a sample for PCR confirmation. Negative days indicate that the sample was collected before the reference PCR confirmation of infection (herein referred as “infection”), whereas positive days represent days following infection. All samples were selected in compliance with the dispositions reported in the clinical protocol and based on patient consent for use of serum.

### Immunosurveillance analysis.

Paired serum samples collected before (2013–2014) and after (2017–2018) ZIKV introduction in the Americas from 1,283 healthy subjects who had participated in the CYD15 Phase III efficacy clinical trial (immunogenicity subset) of the Sanofi Pasteur CYD-TDV vaccine in Colombia, Honduras, Mexico, and Puerto Rico were used to compare Zika serostatus as determined by the combination of Zika MN and Zika BOB results.

### Statistical analysis.

Specificity of Zika BOB and Zika MN was calculated based on the percentage of VCZ samples collected before ZIKV infection that were below the cutoff titers (10 or 100) evaluated. Sensitivity of both assays, on the other hand, was calculated as percentage of VCZ samples collected after ZIKV infection that were above the cutoff titers (10 and 100) evaluated. Parametric unpaired Student’s *t*-test was performed to compare the averages of days post-ZIKV infection between MN/BOB double-positive and MN-positive BOB-negative samples from the VCZ study. Fisher’s exact test was carried out to calculate the odds ratio of MN/BOB double-positive samples to be collected before 180 days post-ZIKV infection. For both analyses, Prism version 7.02 (GraphPad Software Inc., La Jolla, CA) statistical package was used. Differences between groups were deemed significant if *P* < 0.05. The CI for geometric mean titers was within 95%.

## RESULTS

Performance of Zika NS1 BOB ELISA in Clinical Samples from Subjects with VCZ Infection and Comparison with Zika MN.

Longitudinal samples from the phase III efficacy trial CYD15 carried out in Latin America were used in this analysis (Table 1). The selected subjects (*n* = 97) were on average (min, max) 12 (9, 17) years old, 55% female (*n* = 53), who had a detectable ZIKV infection by PCR (VCZ) in 2016. The majority of these individuals (74 of 97) had positive dengue NS1 IgG titers in samples collected before ZIKV infection (pre-Zika). These samples were considered immune to DENV before ZIKV exposure (Table 1).

**Table 1:** Dengue serostatus of the CYD-TDV study participants with virologically confirmed ZIKV infection

Variables	Number of individuals (%*)
Total	97 (100%)
Dengue serostatus	
Naive	9 (9%)
Immune	74 (76%)
Unknown†	14 (14%)
Number of bleed(s) per subject	
1	16 (16%)
2	31 (32%)
3	22 (23%)
≥ 4	28 (29%)

ZIKV = Zika virus.

* Percentage was calculated based on the total number of volunteers analyzed.

† Samples were classified as unknown because of lack of samples before ZIKV infection available for dengue nonstructural protein 1 IgG evaluation.

Zika microneutralization assay and Zika BOB were performed on the pre- (*n* = 78) and post- (*n* = 202) ZIKV infection samples, and the kinetics of antibody production are shown in Figure 1A and B, respectively. The specificity of Zika MN and Zika BOB was 71% and 97%, respectively, and the sensitivity of Zika MN and Zika BOB was 99% and 78%, respectively, when a cutoff titer of 10 was applied to both methods (Table 2). However, the specificity of Zika MN increased to 100% while maintaining a very high sensitivity of 98% when a cutoff titer of 100 was used (Table 2). Of note, multiple samples following ZIKV infection were used from the same subject in this analysis. Unlike the Zika MN, the sensitivity of the Zika BOB was influenced by the timing of sample collection post-infection as it reached 86% on samples collected at a median of 130.5 days (1st/2nd bleeds; mostly from a single sample per subject) and declined to 74% and 65% in samples collected at 220 days (3rd bleed) and 298 days (≥ 4th bleed), respectively (Table 3). A similar trend was also observed in relation to Zika BOB geometric mean titers (Table 3).

**Table 2:** Specificity and sensitivity analysis of Zika MN and Zika BOB immunoassays on virologically confirmed Zika virus cases pre- and post-infection

Assay (cutoff titer)	Specificity			Sensitivity		
	Pre-Zika samples (*N*)	Number negative	% (95% CI)	Post-Zika samples (*N*)	Number positive	% (95% CI)
Zika BOB (10)	78	76	97% (91, 100)	202	157	78% (71, 83)
Zika MN (10)		55	71% (59, 80)		200	99% (97, 100)
Zika MN (100)		78	100% (95, 100)		198	98% (95, 99)

BOB = blockade-of-binding; MN = microneutralization assay.

**Table 3:** Comparison of Zika antibody titers in samples collected in different time points pre- and post-Zika infection

Sample groups	*N*	Days relative to polymerase chain reaction			Zika MN		Zika BOB	
		Min	Max	Median	% of samples MN titers > 100 (sensitivity*)	GMT† (95% CI)	% of samples BOB titers ≥ 10 (sensitivity*)	GMT† (95% CI)
Pre-Zika	78	−1,343	−15	−165	NA*	7.7 (6.5, 9.1)	NA*	5.2 (4.9, 5.5)
1st/2nd bleeds post-Zika	100	17	533	130.5	98%	613.9 (483.0, 780.2)	86%	33.4 (26.5, 41.9)
3rd bleed post-Zika	50	54	605	220	100%	507.4 (389.5, 660.9)	74%	21.7 (15.8, 29.7)
≥ 4th bleed post-Zika	52	119	455	298	100%	455.4 (362.4, 572.3)	65%	16.8 (12.8, 22.0)

BOB = blockade-of-binding; MN = microneutralization assay; GMT = geometric mean titer.

* Sensitivity was calculated with post-Zika samples only.

† For calculation purposes, samples with titers below minimum sample dilution (1:10) were assigned a titer of 5.0.

Next, sample classification agreement was evaluated taking into consideration the cutoff titers that yielded optimal specificity and sensitivity for both assays (100 for Zika MN and 10 for Zika BOB). Samples with titers at or above the cutoff were considered positive and titers below the cutoff were considered negative. As shown in Figure 1C, the MN/BOB double-negative group contained almost entirely pre-Zika samples (76/79) and represents individuals naive to ZIKV. Samples that were MN-negative BOB-positive (*N* = 3) were heterogeneous, comprising both pre- and post-Zika samples and, therefore, classified as undetermined. The MN/BOB double-positive samples consisted entirely (156/156) of post-Zika samples (Figure 1C) collected at an average (95% CI) of 141 (120, 162) days following virus infection (Figure 1D). Samples that were MN-positive BOB-negative also comprised entirely (42/42) post-Zika samples collected at an average (95% CI) of 249 (221, 276) days following virus infection (Figure 1D). Figure 1D shows that MN/BOB double-positive samples were collected at an earlier time point (*P* < 0.0001 with unpaired Student’s *t*-test) than the MN-positive BOB-negative samples, suggesting that Zika BOB likely detects more recent ZIKV exposures. Of note, MN/BOB double-positive samples (75 of 156) were approximately 3-fold (odds ratio 95% CI: 1, 6; *P* = 0.01) more likely to be collected before or at 180 days (6 months) post-ZIKV infection than MN-positive BOB-negative samples (11 of 42).

### False-positive rates of Zika NS1 BOB ELISA in VCD samples.

Samples from repeat DENV exposures have been shown to cause false-positive results on Zika BOB, likely due to cross-reactivity and proximity to the DENV infection.^19^ A panel of serum samples from 98 subjects with a VCD infection from the phase III efficacy trials CYD14 and CYD15, collected in 2012 and 2013, at a median (min, max) of 175 (12, 388) days post-infection, was used to determine the extent these DENV-specific antibodies interfere with the Zika BOB (Table 4). However, DENV serology of these VCD cases before infection was not available to determine the history of virus exposure (primary or secondary infections). As shown in Table 4, 24/98 (24%) VCD samples had positive Zika BOB titers indicating cross-reactivity to DENV infection, which included all four serotypes. Interestingly, VCD samples with positive Zika BOB titers had dengue NS1 IgG levels 7-fold higher than those with negative Zika BOB titers (geometric mean concentration of 5,463 EU/mL and 811 EU/mL, respectively, Supplemental Figure 3).

**Table 4:** False-positive rates of Zika BOB based on samples collected in 2012 and 2013 from subjects with recent virologically confirmed DENV infection

Variables	Number of individuals/samples		Zika BOB GMT† (95% CI)
	Total	False positive (%*)	
Total	98	24%	7.28 (6.28, 8.43)
Time post-DENV infection (days)			
≤ 180	51	27%	7.84 (6.26, 9.82)
> 180	47	19%	6.71 (5.54, 8.14)
Infecting DENV serotype			
DENV-1	45	11 (24%)	7.75 (5.98, 10.04)
DENV-2	27	9 (33%)	7.73 (5.90, 10.13)
DENV-3	20	4 (20%)	6.51 (4.99, 8.53)
DENV-4	5	0 (0%)	5.00 (5.00, 5.00)
Multiple serotypes‡	1	0 (0%)	5.00 (N/A§)

BOB = blockade-of-binding; DENV = dengue virus; GMT = geometric mean titer.

* Percentage was calculated in relation to the total number of individuals in each subgroup (DENV-1, DENV-2, etc.).

† For calculation purposes, samples with titers below the minimum sample dilution (1:10) were assigned a titer of 5.0.

‡ One subject was diagnosed with multiple infections, by DENV-1 and DENV-2.

§ Confidence interval was not calculated because only one value was available for that group.

### Zika immunosurveillance using Zika NS1 BOB ELISA and Zika MN.

To evaluate the performance of Zika BOB and ZIKV MN as immunosurveillance tools to determine Zika serostatus in DENV-endemic areas and to differentiate possible unapparent recent and remote ZIKV exposure, we screened paired samples from the same healthy individuals collected from the immunogenicity subset of the CYD15 Phase III efficacy trial in Colombia, Honduras, Mexico, and Puerto Rico before (2013–2014) and after (2017–2018) the introduction of ZIKV in the Americas (Table 5). Zika serostatus was defined using Zika BOB combined with Zika MN as follows: MN-positive BOB-negative and MN/BOB double-positive results were indicators of remote (> 6 months) and recent (≤ 6 months) virus exposures, respectively. Microneutralization assay/blockade-of-binding double-negative results represented Zika-naive status, whereas MN-negative BOB-positive results were of an undetermined status. The results showed that false-positive Zika serostatus classification in samples collected before ZIKV introduction, whether considered remote or recent, ranged from 1% to 3% or 0% to 1%, respectively, depending on the country of origin (Table 5). By contrast, Zika seropositive rates ranged from 25% to 80% in samples collected at the 2017–2018 time point (Table 5). These samples were collected mostly (20–57%) from recent ZIKV infections, whereas remote exposures accounted for 5–23% of the samples evaluated (Table 5).

**Table 5:** Zika MN and Zika BOB results in samples collected before (2013–2014) and after (2017–2018) ZIKV introduction in the Americas

Countries	*N*	Average age* in years (min, max)	% Female	% of samples							
				Zika serostatus† in 2013–2014				Zika serostatus† in 2017–2018			
				Naive, %	Undeter-mined, %	ZIKV-exposed individuals		Naive, %	Undeter-mined, %	ZIKV-exposed individuals	
						Remote, %	Recent, %			Remote, %	Recent, %
Colombia	703	12 (9, 17)	51	85	10	3	1	45	7	16	32
Honduras	223	12 (9, 17)	49	91	5	2	1	18	2	23	57
Mexico	266	12 (9, 17)	47	90	6	3	0	58	4	7	31
Puerto Rico	91	12 (9, 17)	47	96	2	1	1	75	0	5	20

BOB = blockade-of-binding; MN = microneutralization assay; ZIKV = Zika virus.

* Age information was collected at the time of enrollment.

† Zika serostatus classification was determined based on Zika MN and Zika BOB titers as follows: naive (MN ≤ 100 and BOB < 10), undetermined (MN ≤ 100 and BOB ≥ 10), remote (MN > 100 and BOB < 10), and recent (MN > 100 and BOB ≥ 10) ZIKV exposures.

## DISCUSSION

In this study, we evaluated the performance of two Zika immunoassays, Zika BOB and Zika MN, to detect ZIKV exposures in DENV-endemic areas. The specificity and sensitivity of Zika BOB were 97% and 78% (cutoff titer of 10), respectively, using samples from VCZ cases collected before and after ZIKV infection. In addition, as expected, we found that Zika BOB sensitivity was greater and titers were higher in samples collected at earlier time points post-infection. Thus, unlike the Zika BOB previously described using ZKA35 mAb,^15^ serum antibodies blocking the binding of the F9 mAb are transiently detected in the Zika BOB (within 6 months from the time of infection) and, in line with DENV NS1– and ZIKV NS1–specific IgG3 assays,^2,33^ may represent an alternative tool to identify recent virus exposure. On the other hand, the specificity and sensitivity of Zika MN were 100% and 98%, respectively, at a cutoff titer of 100. Neutralizing antibodies, which have the ability to target multiple epitopes on the virion,^34^ were consistently detected by Zika MN throughout the course of the study and, therefore, cannot distinguish between recent and remote virus exposure. Both assays may be used separately to determine Zika serostatus, even in the background of DENV pre-immunity, although some limitations should be considered: 1) Zika MN is a laborious and relatively low-throughput method that takes 4 days to be completed and requires an infrastructure that may not be widely available in most laboratories, and 2) Zika BOB takes 1 day to be completed, although it needs longitudinal samples collected at least every 6 months, for optimal performance. In addition, Zika BOB results should be confirmed by other specific methods, especially during active DENV outbreaks. Thus, we propose the combination of the results of both methods not only to define Zika serostatus, but also to qualitatively discern recent ZIKV infections as follows: MN/BOB double negative represents samples from ZIKV-naive individuals, MN/BOB double positive may represent samples collected from individuals recently (e.g., ≤ 6 months) exposed to ZIKV, whereas MN positive BOB negative likely represents remote (e.g., > 6 months) ZIKV exposure. Subjects who are MN negative BOB positive are likely false positive to ZIKV infection due to recent DENV infection. Additional studies are necessary to further define the mean duration of recent infection and false-recency rate of BOB titers and establish the duration it remains detectable as previously shown for DENV NS1 IgG3.^33^

Using the Zika serostatus classification shown previously, we also evaluated Zika serostatus in healthy individuals in Colombia, Honduras, Mexico, and Puerto Rico. The subjects were 12 years old on average at the time of enrollment, one of the most susceptible age groups to arbovirus infection in Latin America.^35^ Paired samples were collected from the same individuals (*N* = 1,283) at various time points in 2013–2014 and in 2017–2018. Samples collected in 2013–2014 were shown not to hold significant rates of either recent (≤ 1%) or remote (≤ 3%) ZIKV exposures, despite the occurrence of well-documented DENV outbreaks around the same time period in those countries.^36,37^ Conversely, the frequency of Zika seropositive samples increased as expected in all countries investigated in 2017–2018. Zika seroprevalence in Colombia, Honduras, Mexico, and Puerto Rico was 48%, 80%, 39%, and 25%, respectively, and supports the hypothesis that high levels of herd immunity (decreased number of susceptible naive individuals) are observed in some geographical areas where ZIKV circulated,^2^ impacting disease incidence.^38^ Because the study participants did not report signs or symptoms consistent with ZIKV infection, seropositivity was likely due to unapparent infections. Haby and colleagues in a systematic review and meta-analysis study showed a heterogeneity in the prevalence of unapparent ZIKV infection in the general population (from 29% to 82%),^2^ which corroborates with the wide seroprevalence rates observed in our study. Furthermore, most of the seropositive samples were collected from individuals likely exposed to ZIKV within 6 months from sample collection, which is consistent with the reports of ZIKV transmission in the participating countries in 2016 and 2017.^39^ Moreover, Honduras held the highest rates of remote (> 6 months) ZIKV exposure (23%), followed by Colombia (16%), Mexico (7%), and Puerto Rico (5%), which corresponds to the spatiotemporal spread of ZIKV among these countries from 2015 to 2017.^40^ Factors associated with susceptibility/resistance to ZIKV infection could not be determined in our study because of the lack of longitudinal samples collected shortly before virus exposure. Rodriguez-Barraquer et al.,^2^ however, demonstrated that preexisting immunity to DENV is protective, whereas recent DENV exposures transiently increases susceptibility to ZIKV infection in a community cohort in Salvador, Brazil. Of note, ever since ZIKV was introduced in the Americas, the number of DENV cases, including disease severity and fatalities, decreased substantially until 2017^38^ and started to reemerge in 2018.^41^ Recent ZIKV exposures observed in our study in 2016 and 2017 support the hypothesis of a possible transient cross-immunity between these viruses as previously suggested.^38^

Serological Zika diagnosis is extremely difficult in dengue-endemic areas.^13,16,17^ Zika PRNT offers satisfactory specificity, especially in late convalescent samples,^23^ although its low-throughput and interpretation subjectivity become a liability in high-demand testing enviroments.^24^ The use of Zika BOB along with Zika MN provides an alternative approach to better determine Zika serostatus even in areas where DENV (and other flaviviruses) circulates. In addition, the proposed strategy also potentially allows the differentiation between recent and remote ZIKV exposures, which could be beneficial for calculation of the prevalence and attack rates for immunosurveillance studies.

## Supplemental materials

Supplemental information, table, and figures
