# Supplementary material for: Use of a Blockade-of-Binding ELISA and Microneutralization Assay to Evaluate Zika Virus Serostatus in Dengue-Endemic Areas
Source: Am J Trop Med Hyg. 2019 Aug 6;101(3):708–15. doi: 10.4269/ajtmh.19-0270 (PMC6726926; doi:10.4269/ajtmh.19-0270)
Supplement: Supplementary file 1 [file tpmd190270.SD1.pdf]

Supplemental Tables

Supplemental Table 1. Antigen specificity analysis of the Zika NS1-specific monoclonal antibody (mAb), clone F9, used for Zika NS1 blockade of binding enzyme-linked immunosorbent assay development. Indirect ELISA was carried out by coating NS1 from different flaviviruses. Following blocking, mAb F9 was added to the plate and later detected with a species-specific conjugate. Table shows the binding of the mAb to NS1 from different flaviviruses as the optical density normalized by the signal on the ZIKV NS1.

NS1 Proteins	% OD450 nm of the Zika NS1 Protein
ZIKV	100%
DENV-1	1%
DENV-2	1%
DENV-3	1%
DENV-4	1%
YFV	1%
JEV	1%
TBEV	1%
USUV	17%
WNV	1%

DENV, dengue virus; JEV, Japanese encephalitis virus; OD, optical density; USUV, Usutu virus; WNV, West Nile virus; YFV, yellow fever virus, ZIKV, Zika virus

Supplemental Table 2. Performance characteristics of internal quality controls in the Zika NS1 BOB ELISA and Zika Microneutralization assay.

Immunoassay	Internal quality control	Number of Determinations[3]	GMT	%GCV[1]	Acceptable limits	
					Lower	Upper
Zika NS1 BOB ELISA	Titer high control	231	69.56	19.1%	49.04	98.66
	Titer low control	223	17.94	23.8%	11.71	27.47
	Titer negative control	230	<10.00	N/A[2]	N/A[2]	N/A[2]
	mAb OD %CV	234	12.20%	66.2%	N/A	<30%
	mAb signal (OD)	234	2.34	25.0%	1.50	3.66
Zika Microneutralization Assay	Titer control 1	45	282	60.6%	141	564
	Titer control 2	48	247	53.3%	123	493
	Titer negative control	34	<10	N/A[2]	N/A[2]	N/A[2]
	TCID50	45	300[4]	43.0%	95	949

CV, coefficient of variance; GCV, geometric coefficient of variation; GMT, geometric mean titer; mAb, monoclonal antibody; OD,optical density

[1]%GCV – coefficient of variance of the geometric mean

[2]N/A – Not calculated because the GMT was below the lower limit of quantitation, or no data was available for calculation

[3]Determinations refer to number of values obtained in the course of multiple weeks to establish valid ranges for each parameter evaluated

[4]GMT of TCID50 consist of a target value

Supplemental Figure

Supplemental Figure S1.

Evaluation of Zika NS1 blockade of binding enzyme-linked immunosorbent assay performance recommended by the ICH Harmonized Tripartite guidelines [31]. (A) Analytical specificity analysis of the monoclonal antibody (mAb, clone F9). Heat-map shows percent inhibition of the binding of the mAb to the Zika NS1 in the microtiter ELISA plate by the homologous and heterologous competitors. Red represents competition over 50%. White represents competition at 50%, while blue represents competition below 50% (B). Matrix effect analysis. Plot shows the geometric mean of the blockade titers a Zika virus positive antibody sample spiked in three concentrations on hemolytic (red circle), icteric (yellow square) and lipidic (orange triangle) matrices. Blue star represent the expected geometric mean of blockade titer of the same sample analyzed at 100%. Table shows the percent recovery (%Rec) of each spike concentration in relation to the expected geometric mean blockade titer (C). Accuracy analysis. Plot shows the geometric mean of the blockade titers of a Zika virus positive antibody sample spiked in four concentrations with normal Zika virus antibody negative serum. Blue stars represent the expected geometric mean of blockade titer of the same sample analyzed at 100%. Table shows the percent recovery (%Rec) of each spike concentration in relation to the expected geometric mean blockade titer (D). Linearity and dilutability analysis. Ten samples with known blockade titers were analyzed in four concentrations covering a wide range of the assay. Plot shows the expected blockade titers versus the obtained blockade titers in each concentration analyzed. Gray dashed line represent the linear regression of samples combined. For each sample, coefficient

of determination and slope were determined. (E). Repeatability analysis Plot shows percent geometric mean coefficient of variation (%GCV) of each measurement by three analysts in samples with a wide range of blockade titer levels. Red dotted line represents the calculated repeatability of 23.0%. (F). Intermediate precision analysis. Plot shows percent geometric mean coefficient of variation (%GCV) of each measurement in each samples by different analysists on multiple days with a wide range of blockade titer levels. Red dotted line represents the calculated intermediate precision of 23.4%. Dashed gray line represents the assay lower limit of quantitation.

[1]Figure E, analyst 3 only tested a subset of the sample panel used to evaluate intra-assay repeatability.

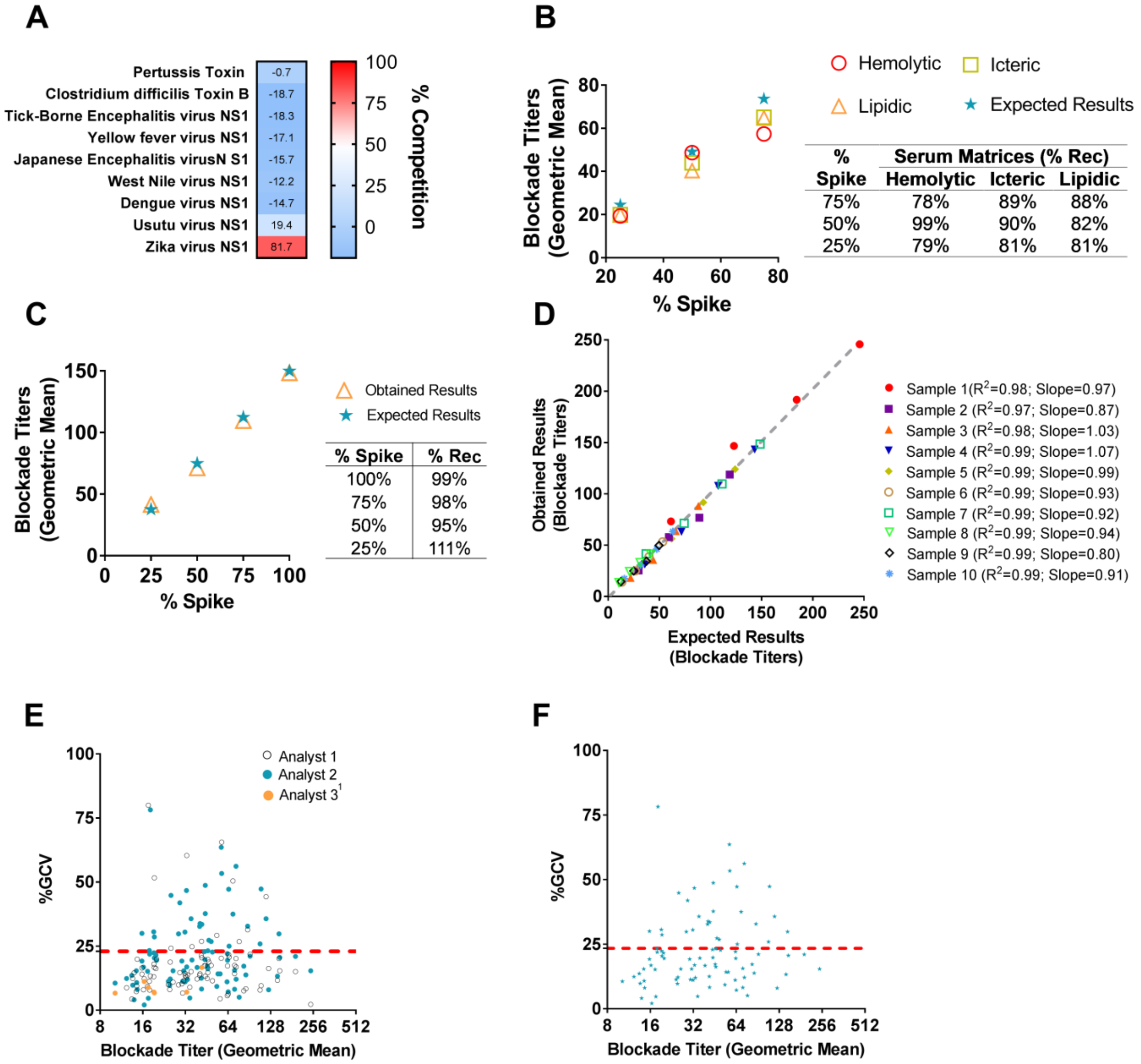
A
Pertussis Toxin -0.7
Clostridium difficilis Toxin B -18.7
Tick-Borne Encephalitis virus NS1 -18.3
Yellow fever virus NS1 -17.1
Japanese Encephalitis virusN S1 -15.7
West Nile virus NS1 -12.2
Dengue virus NS1 -14.7
Usutu virus NS1 19.4
Zika virus NS1 81.7
% Competition
100
75
50
25
0
B
Hemolytic
Icteric
Lipidic
Expected Results
Blockade Titers (Geometric Mean)
% Spike
% Spike | Serum Matrices (% Rec): Hemolytic, Icteric, Lipidic
75% 78% 89% 88%
50% 99% 90% 82%
25% 79% 81% 81%
C
Obtained Results
Expected Results
Blockade Titers (Geometric Mean)
% Spike
% Spike | % Rec
100% 99%
75% 98%
50% 95%
25% 111%
D
Obtained Results (Blockade Titers)
Expected Results (Blockade Titers)
Sample 1(R2=0.98; Slope=0.97)
Sample 2 (R2=0.97; Slope=0.87)
Sample 3 (R2=0.98; Slope=1.03)
Sample 4 (R2=0.99; Slope=1.07)
Sample 5 (R2=0.99; Slope=0.99)
Sample 6 (R2=0.99; Slope=0.93)
Sample 7 (R2=0.99; Slope=0.92)
Sample 8 (R2=0.99; Slope=0.94)
Sample 9 (R2=0.99; Slope=0.80)
Sample 10 (R2=0.99; Slope=0.91)
E
Analyst 1
Analyst 2
Analyst 3
%GCV
Blockade Titer (Geometric Mean)
F
%GCV
Blockade Titer (Geometric Mean)

Supplemental Figure S2. Representative blockade curves (n=36) of a ZIKV-positive or negative antibody sample. The plot depicts the normalized geometric mean signal of the competition between mAb F9 and serial fold dilutions of a serum sample. The mAb F9 signal is normalized to the maximum signal observed in the absence of competing human sera sample. Geometric mean of multiple blockade curves of a Zika-negative- and a Zika-positive antibody sample yielded consistent blockade titers (geometric mean titer [GMT] of the ZIKV antibody positive sample [95% confidence interval, CI] = 67.23 [65.45, 69.05]. Error bars represent the geometric standard deviation. The red dotted line represents 50% inhibition based on the maximum signal.

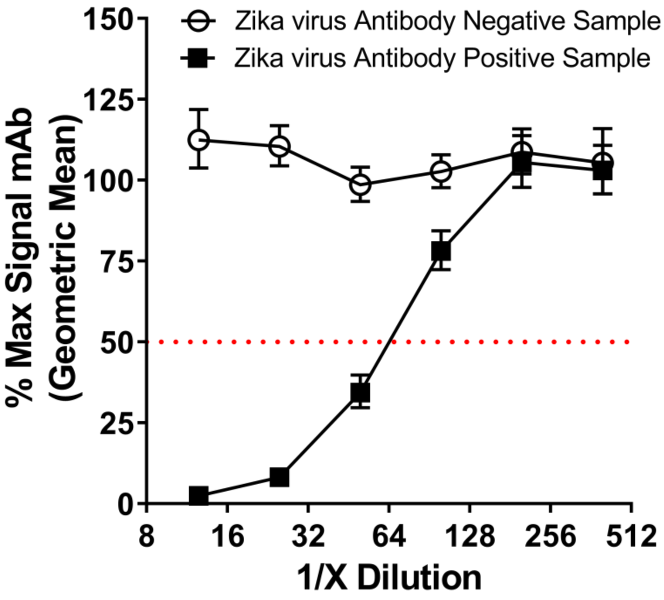

Supplemental Figure S3. Dengue NS1 IgG levels in pre-Zika and virologically confirmed dengue (VCD) samples used to evaluate the specificity of the Zika NS1 BOB ELISA. Plot shows Dengue NS1 IgG levels for individual samples presenting (BOB+) or not (BOB-) Zika NS1 blockade titers. Red lines represent the geometric mean and standard deviation of the geometric mean, respectively. The black dotted line represents the assay lowest limit of quantitation as 9 EU/mL. Samples with dengue NS1 IgG levels < 9 EU/mL were assigned an arbitrary value of 4.5 EU/mL for calculation purposes.

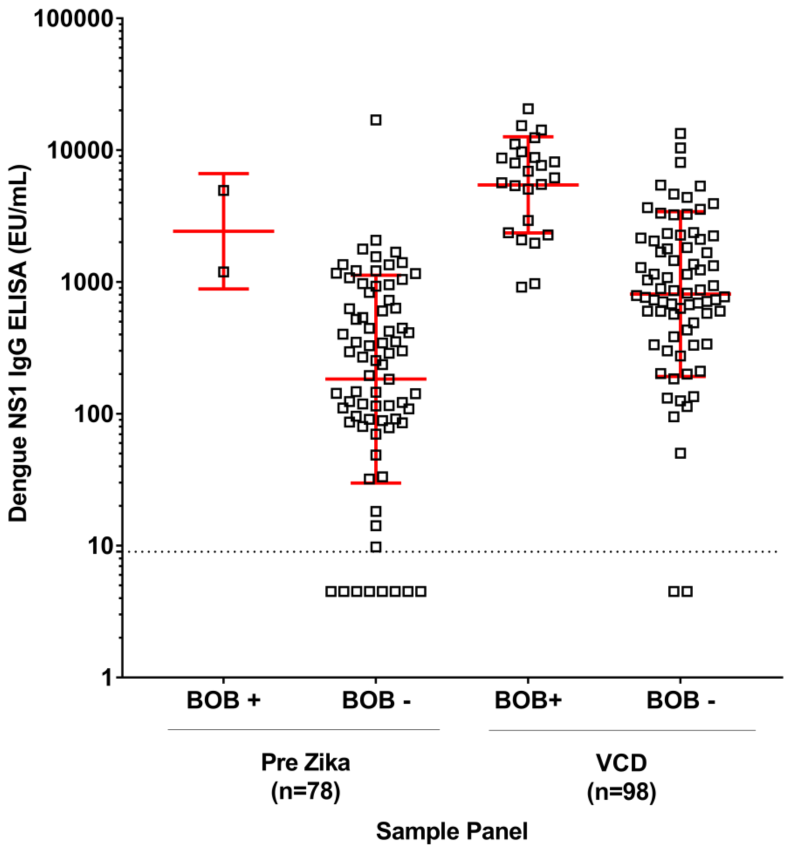

Supplemental Material

Zika Immunoassay Characterization

I. *Antigen Competition (Zika BOB only):* Competition studies were performed using the homologous and heterologous (related competitor: using NS1 from other flaviviruses; and unrelated competitor: *Bordetella pertussis* toxin and *Clostridium difficile* toxin B) competitors at 30µg/mL and mAb. Specificity is assessed as percent competition, calculated as [1-(Optical Density of the Competed mAb ÷ Optical Density of the Uncompeted mAb)] × 100%.

II. *Serum Competition (Zika MN only):* Anti-ZIKV positive human serum samples were spiked with samples containing known antibodies to: Yellow Fever virus (YFV), Japanese Encephalitis virus (JEV), West Nile virus (WNV) and/or Dengue virus (DENV) and anti-ZIKV negative human serum (baseline control). Percentage of samples with percent recovery within ± 50% of the expected value for each spiked sample was then calculated.

III. *Matrix Effect:* Spike recovery of characterized ZIKV antibody positive samples available commercially (ABO Pharmaceuticals, San Diego, USA) diluted into hemolytic (Rockland, Limerick, USA), lipidic (Calbiochem, Temecula, USA), icteric (Calbiochem, Temecula, USA) matrices. For Zika BOB, ZIKV samples were prepared at 75% (v/v), 50% (v/v) and 25% (v/v). Percent recovery (%Rec) is calculated as (Observed Result ÷ Expected Result) × 100%. *For Zika MN, ZIKV samples were prepared only at 50% (v/v) in each matrix.* Percentage of samples with percent recovery within ± 50% of the expected value for each spiked sample was then calculated.

IV. Accuracy: Spike recovery of characterized ZIKV antibody positive samples available commercially (ABO Pharmaceuticals, San Diego, USA) into ZIKV antibody negative matrices. For Zika BOB, %Rec was calculated as shown above. For Zika MN, the percentage of samples with percent recovery within ± 50% of the expected GMT was calculated.

V. Precision: Precision of the assay was assessed using a panel of 100 ZIKV antibody positive samples (from commercial source [ABO Pharmaceuticals, San Diego, USA] and CYD15 phase III efficacy clinical trial) spanning a wide range of concentrations and tested by multiple analysts to generate replicate results within runs (repeatability), as well as across runs (intermediate precision). Both repeatability and intermediate precision are assessed using the geometric coefficient of variation (GCV) expressed as a percentage, %GCV, for both Zika BOB and Zika MN.

VI. LLOQ Establishment and Verification: The minimum concentration at which samples yielded determinations with suitable precision and accuracy was established as the LLOQ. The established LLOQ was challenged and verified using a panel of ZIKV antibody positive samples (ABO Pharmaceuticals, San Diego, USA) with concentrations near the LLOQ of the assay for both Zika BOB and Zika MN.

VII. Linearity or Dilutability: For both Zika BOB and Zika MN, dilutability was assessed based on spike recovery of characterized ZIKV antibody positive samples (from commercial source [ABO Pharmaceuticals, San Diego, USA] and/or CYD15 phase III efficacy clinical trial) tested as neat (undiluted) and at

least three prepared dilutions. Linearity was calculated by plotting the expected result as the independent variable (x-axis) and the observed result as the dependent variable (y-axis) and fitting a linear regression. The slope and coefficient of determination (R^2) for the linear regression was used for evaluating dilutability or linearity.

Zika Immunoassays Characterization Results

I. **Zika NS1 BOB ELISA:** Supplemental Figure S3 shows the evaluation of the assay based on the ICH Harmonized Tripartite [31] and Clinical Laboratory and Standards Institute's EP-17 [43] guidelines, as detailed in the methods section. Antigen analytical specificity for mAb F9 demonstrated that, within the expected variability margin for mAb binding to the coated antigen, homologous antigens inhibited over 80% of the mAb signal, while heterologous competition was ≤ 20% (Figure S1A). Thus, the mAb F9 is considered specific to ZIKV NS1 protein. Moreover, ZIKV antibody-positive spiked samples into hemolytic, icteric or lipidic matrices demonstrated percent recovery of 85%, 87% and 84%, respectively (Figure S1B), suggesting the matrices evaluated did not interfere with the performance of the assay. Accuracy analysis of a sample panel with established blockade titers at different concentrations was carried out and yielded %Rec that ranged from 95% to 111% (Figure S1C). Assay linearity was evaluated using 10 samples, with established blockade titers at 4 concentrations. Coefficient of determination (R^2) and slope for all curves ranged from 0.97 to 0.99 and 0.80 to 1.07, respectively (Figure S1D). Repeatability of the assay was evaluated using

100 samples, tested 5 times by 3 different analysts per individual run. The percent geometric coefficient of variation (%GCV) for repeatability is shown in Figure S1E. The overall assay repeatability was 23.0% (95% CI 21.8%, 24.4%), which is lower than what is the accepted range for titer-based functional assays (< 50%). Moreover, the variability of the assay taking into account different analysts performing the assay on multiple days and using different instruments was 23.4% (95% CI 21.2%, 25.7%) (Figure S1F), which within the accepted range for titer-based functional assays (< 50% GCV). The LLOQ was verified using ZIKV-positive samples with a known blockade titer ranging from below to up to 4-fold higher than the minimum sample dilution (1:10). The results indicate that among all concentrations tested, the assay intermediate precision at LLOQ level was estimated as 30.4% (95% CI 26.0%, 36.8%) (Supplemental Table 3).

II. **ZIKV Microneutralization Assay:** Assay specificity is summarized in Supplemental Table 4. The percentage of samples with observed GMT within ± 50% of the expected value for each spiked sample were 90.0% (9/10) for YFV-spiked samples, 90.0% (9/10) for JEV-spiked samples, 100.0% (10/10) for WNV-spiked samples and 90.0% for DEN Sample 1-spiked samples. However, DENV positive samples 2, demonstrated 40.0% (4/10) and 20.0% (2/10) of the samples, respectively, exhibited percent differences (expected versus observed GMT) within the range ± 50.0% of expected GMT. These results indicate that the ZIKV MN assay exhibits potential cross-reactivity with sera containing anti-DENV antibodies, but not with antibodies to other tested flaviviruses (YFV, JEV and WNV). The results of the serum matrix effect study to evaluate ZIKV MN assay

specificity are summarized in Supplemental Table 5. Results shows that 80.0% (8/10), 90.0% (9/10) and 100.0% (10/10) of the samples had the percent difference within ± 50% of samples spiked with icteric, hemolytic and lipemic matrices, respectively. These results indicate that samples tested in the ZIKV MN assay are not affected by serum matrix interferents. The results of dilutional accuracy and dilutability are summarized in Supplemental Table 6. Sample 10 diluted 1:20 had an expected GMT less than LLOQ (10) and, thus, excluded from analysis. In addition, only one valid result was obtained for undilute sample 10. Sample 10, undilute was included in the statistical analysis. The estimation of expected value for Sample 10 dilutions may have been significantly impacted by the variation of the assay. The percentage of samples with observed GMT within ± 50% of the expected GMT were 90.0% (9/10) for dilutions at 1:5, 80.0% (8/10) for dilutions at 1:10 and 88.9% (8/9) for dilutions at 1:20. Overall, 86.2% (25/29) of samples/dilutions had observed GMT with ± 50% of the expected GMT. The Intra-assay precision results were generated by testing 42 human serum samples with Zika antibody titers that cover the range of the assay, five times each in a single assay run. The overall %GCV was 54.4% with a 95% confidence interval of (50.5%, 58.8%) which was within the expected precision of 60% GCV. A precision profile plot for repeatability is shown in Supplemental Figure 3. Intra-assay precision for ZIKV MN assay is established as %GCV ≤ 60%.Intermediate Precision was determined by testing 42 human serum samples in 3 independent assay runs by at least two different analysts. The overall %GCV for intermediate precision was 55.3% with a 95% confidence interval of (51.0%, 60.3%) which

was within the expected precision of 60% GCV. A precision profile plot for intermediate precision is shown in Supplemental Figure S4. Intermediate precision for ZIKV MN assay is established as %GCV ≤ 60%.The results of LLOQ determination are summarized in Supplemental Table 7. Statistical analysis showed that for intra-assay precision (repeatability) near LLOQ, the overall %GCV for positive samples with GMT near the expected LLOQ (i.e., 10-40) was 55.1% with a 95% confidence interval of (48.2%, 64.1%) which was within the expected precision of 60% GCV. For intermediate precision near LLOQ, the overall %GCV for positive samples with GMT near the expected LLOQ (i.e., 10-40) was 55.3% with a 95% confidence interval of (48.4%, 64.4%) which was within the expected precision of 60% GCV.

Supplemental Figure S4: Profile Plot for Precision for Zika MN

[A] Repeatability [B] Intermediate Precision

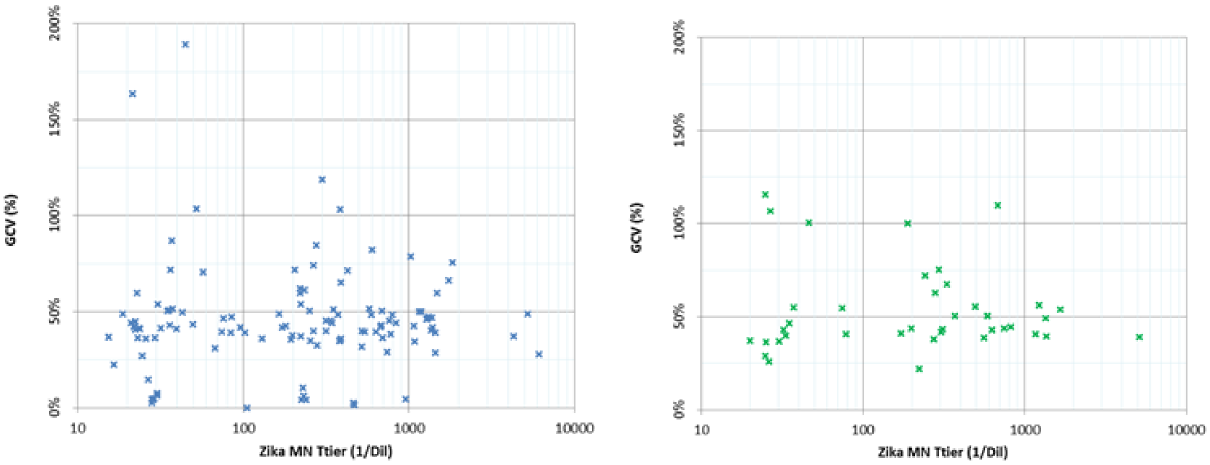

Supplemental Table 3. Evaluation of the lower limit of quantitation of the Zika NS1 blockade of binding enzyme-linked immunosorbent assay.

Sample ID	GMT	CV (%)			CV (%) (Overall)
		Analyst 1	Analyst 2	Analyst 3	
Sample 1	<10	N/D[1]	N/D[1]	N/D[1]	N/D[1]
Sample 2	<10	N/D[1]	N/D[1]	N/D[1]	N/D[1]
Sample 3	10.2	10.6%	N/D[1]	6.7%	38.7%
Sample 4	<10	N/D[1]	N/D[1]	N/D[1]	N/D[1]
Sample 5	16.4	2.1%	12.3%	11.2%	13.3%
Sample 6	17.5	4.7%	80.0%	8.9%	45.3%
Sample 7	19.3	28.6%	51.7%	6.8%	39.2%
Sample 8	19.2	22.4%	18.0%	7.1%	15.1%
Sample 9	32.7	16.9%	60.4%	7.1%	33.3%
Sample 10	41.8	33.5%	20.5%	16.7%	27.9%

CV, coefficient of variance; GMT, geometric mean titer

[1]N/D – Not determined because the GMT is <lower limit of quantitation or no data available for calculation

Supplemental Table 4: Summary of ZIKV MN Assay Specificity by Serum Spiking

Sample ID	Matrix	Results (1/Dilution)			Geometric Mean Titer (1/Dil)	% Difference (Observed vs. Expected GMT)
		Replicate #1	Replicate #2	Replicate #3		
Sample 1	Negative Sample	56	57	37	49.1	ND
	YFV Sample	57	58	UTDT	57.5	17.2%
	JEV Sample	57	51	61	56.2	14.5%
	WNV Sample	56	58	58	57.3	16.8%
	DEN-Sample 1	58	29	28	36.1	-26.4%
	DEN-Sample 2	109	110	213	136.7	178.6% ‡
	DEN-Sample 3	UTDT	394	UTDT	394.0	703.0% ‡
Sample 2	Negative Sample	224	223	223	223.3	ND
	YFV Sample	229	216	220	221.6	-0.8%
	JEV Sample	230	116	229	182.8	-18.1%
	WNV Sample	239	227	232	232.6	4.2%
	DEN-Sample 1	118	235	116	147.6	-33.9%
	DEN-Sample 2	453	433	UTDT	442.9	98.3% ‡
	DEN-Sample 3	905	257	800	570.9	155.6% ‡
Sample 3	Negative Sample	243	229	229	233.6	ND
	YFV Sample	234	184	230	214.7	-8.1%
	JEV Sample	438	177	228	260.5	11.5%
	WNV Sample	154	208	454	244.1	4.5%
	DEN-Sample 1	227	212	146	191.5	-18.0%
	DEN-Sample 2	446	444	460	449.9	92.6% ‡
	DEN-Sample 3	1493	855	902	1048.1	348.7% ‡
Sample 4	Negative Sample	920	431	456	565.5	ND
	YFV Sample	456	216	201	270.5	-52.2% ‡

Sample ID	Matrix	Results (1/Dilution)			Geometric Mean Titer (1/Dil)	% Difference (Observed vs. Expected GMT)
		Replicate #1	Replicate #2	Replicate #3		
	JEV Sample	460	877	473	575.7	1.8%
	WNV Sample	466	891	472	580.9	2.7%
	DEN-Sample 1	393	248	449	352.4	-37.7%
	DEN-Sample 2	952	839	879	888.8	57.2% ‡
	DEN-Sample 3	428	3322	UTDT	1192.4	110.9% ‡
Sample 5	Negative Sample	954	882	UTDT	917.3	ND
	YFV Sample	UTDT	1676	898	1226.8	33.7%
	JEV Sample	1722	UTDT	902	1246.3	35.9%
	WNV Sample	1816	841	UTDT	1235.8	34.7%
	DEN-Sample 1	966	1747	892	1146.1	24.9%
	DEN-Sample 2	428	464	1678	693.3	-24.4%
	DEN-Sample 3	754	1761	UTDT	1152.3	25.6%
Sample 6	Negative Sample	<10	<10	<10	<10	ND
	YFV Sample	<10	<10	<10	<10	N/A §
	JEV Sample	<10	<10	<10	<10	N/A §
	WNV Sample	<10	<10	<10	<10	N/A §
	DEN-Sample 1	<10	<10	<10	<10	N/A §
	DEN-Sample 2	56	15	27	28.3	N/A §
	DEN-Sample 3	53	66	27	45.5	N/A §
Sample 7	Negative Sample	217	220	104	170.6	ND
	YFV Sample	220	216	117	177.2	3.8%
	JEV Sample	UTDT	223	224	223.5	31.0%
	WNV Sample	211	UTDT	UTDT	211.0	23.7%
	DEN-Sample 1	103	112	105	106.6	-37.5%
	DEN-Sample 2	215	219	208	214.0	25.4%
	DEN-Sample 3	212	249	437	284.7	66.9% ‡
Sample	Negative	54	54	106	67.6	ND

Sample ID	Matrix	Results (1/Dilution)			Geometric Mean Titer (1/Dil)	% Difference (Observed vs. Expected GMT)
		Replicate #1	Replicate #2	Replicate #3		
8	Sample					
	YFV Sample	83	103	107	97.1	43.6%
	JEV Sample	111	108	52	85.4	26.3%
	WNV Sample	54	107	150	95.3	41.0%
	DEN-Sample 1	110	110	107	109.0	61.2% ‡
	DEN-Sample 2	456	218	234	285.5	322.2% ‡
	DEN-Sample 3	438	UTDT	894	625.8	825.5% ‡
Sample 9	Negative Sample	217	228	107	174.3	ND
	YFV Sample	222	110	77	123.4	-29.2%
	JEV Sample	113	104	110	108.9	-37.5%
	WNV Sample	109	106	225	137.5	-21.1%
	DEN-Sample 1	123	107	110	113.1	-35.1%
	DEN-Sample 2	425	221	217	273.2	56.7% ‡
	DEN-Sample 3	218	236	223	225.5	29.4%
Sample 10	Negative Sample	438	465	220	355.2	ND
	YFV Sample	219	419	204	265.5	-25.2%
	JEV Sample	441	214	UTDT	307.2	-13.5%
	WNV Sample	UTDT	440	350	392.4	10.5%
	DEN-Sample 1	143	458	222	244.1	-31.3%
	DEN-Sample 2	220	UTDT	446	313.2	-11.8%
	DEN-Sample 3	452	914	UTDT	642.8	81.0% ‡
Sample 11	Negative Sample	435	534	433	465.1	ND
	YFV Sample	221	963	915	579.6	24.6%

Sample ID	Matrix	Results (1/Dilution)			Geometric Mean Titer (1/Dil)	% Difference (Observed vs. Expected GMT)
		Replicate #1	Replicate #2	Replicate #3		
	JEV Sample	444	913	844	699.4	50.4% ‡
	WNV Sample	447	423	912	556.6	19.7%
	DEN-Sample 1	456	303	434	391.4	-15.8%
	DEN-Sample 2	678	841	492	654.6	40.8%
	DEN-Sample 3	1800	854	1814	1407.5	202.7% ‡

‡Percent recovery >50%

§N/A, not applicable as observed GMT was <10

UTDT – Unable to be determined

ND – Not determined because only observed values were determined

Supplemental Table 5: Summary of ZIKV MN Assay Specificity by Matrix Effect

Sample ID	Matrix	Results (1/Dil)			Geometric Mean Titer (1/Dil)	% Difference (Observed vs. Expected GMT)
		Replicate #1	Replicate #2	Replicate #3		
Sample 1	Negative	58	28	57	45.2	ND
	Hemolytic	62	56	57	58.3	28.8%
	Icteric	60	57	27	45.2	-0.1%
	Lipemic	56	59	58	57.7	27.4%
Sample 2	Negative	233	230	111	181.2	ND
	Hemolytic	242	107	224	179.7	-0.8%
	Icteric	195	170	124	160.2	-11.6%
	Lipemic	230	117	227	182.8	0.9%
Sample 3	Negative	489	328	222	329.0	ND
	Hemolytic	238	472	478	377.3	14.7%
	Icteric	475	222	471	367.6	11.7%
	Lipemic	UTDT†	461	120	235.2	-28.5%
Sample 4	Negative	918	470	928	737.0	ND
	Hemolytic	924	481	950	750.2	1.8%
	Icteric	966	921	942	942.8	27.9%
	Lipemic	475	912	515	606.5	-17.7%
Sample 5	Negative	832	1843	1798	1402.2	ND
	Hemolytic	UTDT	920	1850	1304.6	-7.0%
	Icteric	1083	983	936	998.8	-28.8%
	Lipemic	973	904	1877	1181.9	-15.7%
Sample 6	Negative	<10	<10	<10	<10	ND
	Hemolytic	<10	<10	<10	<10	N/A ‡
	Icteric	<10	<10	<10	<10	N/A ‡
	Lipemic	<10	<10	<10	<10	N/A ‡
Sample 7	Negative	124	228	461	235.3	ND
	Hemolytic	237	UTDT	228	232.5	-1.2%

Sample ID	Matrix	Results (1/Dil)			Geometric Mean Titer (1/Dil)	% Difference (Observed vs. Expected GMT)
		Replicate #1	Replicate #2	Replicate #3		
	Icteric	474	221	UTDT	323.7	37.5%
	Lipemic	119	197	211	170.4	-27.6%
Sample 8	Negative	234	223	110	179.0	ND
	Hemolytic	225	226	230	227.0	26.8%
	Icteric	229	223	115	180.4	0.8%
	Lipemic	117	114	116	115.7	-35.4%
Sample 9	Negative	UTDT	109	113	111.0	ND
	Hemolytic	241	217	UTDT	228.7	106.1% §
	Icteric	241	218	228	228.8	106.2% §
	Lipemic	231	138	233	195.1	75.8%
Sample 10	Negative	UTDT	460	234	328.1	N/A
	Hemolytic	882	919	894	898.2	173.8% §
	Icteric	111	588	454	309.4	-5.7%
	Lipemic	460	280	644	436.1	32.9%
Sample 11	Negative	441	946	451	573.0	ND
	Hemolytic	UTDT	440	457	448.4	-21.7%
	Icteric	474	442	920	577.6	0.8%
	Lipemic	495	893	484	598.1	4.4%

‡N/A, not applicable as observed GMT was < 10
§Percent difference > 50%
ND – Not determined because only observed values were determined

Supplemental Table 6: Summary of Dilutability of Individual Samples for ZIKV MN Assay

Sample ID	Dilution	Results (1:Dilution)					Expected GMT	Observed GMT	% Difference (Observed vs. Expected GMT)
		Rep #1	Rep #2	Rep #3	Rep #4	Rep #5			
Sample 1	Undilute	UTDT	1223	1793	1785	1109	N/A	1443.4	ND
	1/5	377	462	231	285	227	288.7	304.2	5.4%
	1/10	225	226	192	122	86	144.3	159.3	10.3%
	1/20	102	UTDT	118	114	58	72.2	94.5	30.9%
Sample 2	Undilute	1806	1446	1854	910	<10	N/A	1448.8	ND
	1/5	220	233	235	230	217	289.8	226.9	-21.7%
	1/10	110	116	57	56	UTDT	144.9	79.9	-44.9%
	1/20	56	58	58	29	57	72.4	50.0	-31.0%
Sample 3	Undilute	985	1850	1705	923	1838	N/A	1394.4	ND
	1/5	229	227	228	121	822	278.9	259.6	-6.9%
	1/10	114	98	116	114	457	139.4	146.5	5.1%
	1/20	57	32	58	35	114	69.7	53.1	-23.8%
Sample 4	Undilute	1848	1802	919	1806	886	N/A	1374.0	ND
	1/5	120	228	225	231	217	274.8	198.6	-27.7%
	1/10	114	121	111	114	114	137.4	114.8	-16.5%
	1/20	56	60	56	56	56	68.7	56.8	-17.4%
Sample 5	Undilute	1818	915	934	UTDT	1751	N/A	1284.3	ND
	1/5	399	231	226	122	225	256.9	224.6	-12.6%

Sample ID	Dilution	Results (1:Dilution)					Expected GMT	Observed GMT	% Difference (Observed vs. Expected GMT)
		Rep #1	Rep #2	Rep #3	Rep #4	Rep #5			
	1/10	57	113	114	54	115	128.4	85.5	-33.5%
	1/20	58	58	30	54	57	64.2	49.9	-22.2%
Sample 6	Undilute	7274	UTDT	3667	3704	7374	N/A	5195.3	ND
	1/5	1882	1386	912	904	928	1039.1	1148.2	10.5%
	1/10	138	170	427	UTDT	238	519.5	221.0	-57.5% ‡
	1/20	234	181	148	UTDT	229	259.8	194.6	-25.1%
Sample 7	Undilute	231	122	112	233	UTDT	N/A	164.7	ND
	1/5	60	54	56	34	29	32.9	44.7	35.8%
	1/10	29	28	UTDT	28	29	16.5	28.5	73.0% ‡
	1/20	16	14	14	<10	13	8.2	14.2	72.6% ‡
Sample 8	Undilute	118	246	451	444	230	N/A	266.2	ND
	1/5	65	60	56	57	58	53.2	59.1	11.0%
	1/10	30	58	28	29	29	26.6	33.3	25.1%
	1/20	16	15	14	14	29	13.3	16.9	26.7%
Sample 9	Undilute	1825	859	UTDT	906	931	N/A	1072.3	ND
	1/5	233	155	235	118	237	214.5	188.4	-12.2%
	1/10	57	103	58	56	UTDT	107.2	66.1	-38.4%
	1/20	59	30	61	22	70	53.6	44.1	-17.8%

Sample ID	Dilution	Results (1:Dilution)					Expected GMT	Observed GMT	% Difference (Observed vs. Expected GMT)
		Rep #1	Rep #2	Rep #3	Rep #4	Rep #5			
Sample 10*	Undilute	UTDT	UTDT	UTDT	UTDT	106	N/A	106.0	ND
	1/5	28	58	34	58	30	21.2	39.5	86.3% ‡
	1/10	13	14	14	15	14	10.6	14.0	31.9%
	1/20	24	<10	14	13	<10	<10	10.2	N/A

‡Percent recovery > 50%

*Sample 10 diluted 1:20 had an expected GMT less than LLOQ (10) and, thus, excluded from analysis. In addition, only one valid result was obtained for undilute sample 10. Sample 10, undilute was included in the statistical analysis. The estimation of expected value for Sample 10 dilutions may have been significantly impacted by the variation of the assay.

UTDT – Unable to be determined

ND – Not determined because only observed values were determined

N/A – Not applicable as observed GMT was < 10

Supplemental Table 7: Summary Intra- and Inter-mediate Precision of ZIKV MN Assay on LLOQ samples

Sample ID	Intra-of Run #1 of the Sample		Intra- of Run #2 of the Sample		Intra- of Run #3 of the Sample		Intermediate Precision of the Sample	
	GMT (1/Dil)	GCV%	GMT (1/Dil)	GCV%	GMT (1/Dil)	GCV%	GMT (1/Dil)	GCV%
Sample 1	21.5	163.4%	22.8	59.8%	37.2	87.0%	26.6	106.9%
Sample 2	28.2	4.7%	30.3	7.7%	18.7	49.0%	25.2	36.3%
Sample 3	39.5	41.2%	35.1	50.6%	30.5	53.9%	34.8	46.7%
Sample 4	29.3	36.5%	35.9	43.1%	35.5	50.7%	33.1	40.0%
Sample 5	28	2.6%	22.4	45.0%	28.6	4.1%	26.2	25.9%
Sample 6	31.8	41.5%	36.2	71.7%	29	4.9%	32.2	42.9%
Sample 7	42.7	49.6%	25.9	36.0%	49.6	43.6%	37.3	55.0%
Sample 8	23.2	36.4%	21.1	44.1%	16.5	22.6%	20.1	37.1%
Sample 9	37.4	51.5%	30.2	6.5%	24.7	27.2%	30.3	36.8%
Sample 10	26.6	14.8%	23.5	41.4%	23.8	41.2%	24.8	29.0%
Sample 11	22.3	40.7%	44.8	189.0%	15.5	36.7%	24.9	115.7%
